# Retraction: Rescue of MHC-1 Antigen Processing Machinery by Down-Regulation in Expression of IGF-1 in Human Glioblastoma Cells

**DOI:** 10.1371/journal.pone.0305595

**Published:** 2024-06-12

**Authors:** The PLOS ONE Editors

After this article [1] was published, concerns were raised about Figures 4, 5 and 7.

Specifically:

- The TAP-1 panel in Figure 4A appears similar to the LMP-7 panel in Figure 4C.
- There appear to be vertical discontinuities between lanes M and 1 and between lanes 1 and 2 suggestive of splicing in Figure 4B. In addition, lanes 1 and 2 appear similar.
- Lanes 1–5 of the Actin panel in Figure 5A appear similar to lanes 2–6 of the β-Actin panel in Figure 7A, with altered exposure.

The authors did not respond to requests for comment on the above concerns. In the absence of the original blots, gels and data underlying the published figures, and in light of the concerns affecting multiple figures which cast doubt on the reliability of the results, the *PLOS ONE* Editors retract this article.

All authors either did not respond directly or could not be reached.

## References

[1] Pan Y, Trojan J, Guo Y, Anthony DD (2013) Rescue of MHC-1 Antigen Processing Machinery by Down-Regulation in Expression of IGF-1 in Human Glioblastoma Cells. PLoS ONE 8(3): e58428. doi: 10.1371/journal.pone.0058428 23526983 PMC3603982

